# Minimal important difference after hand surgery: a prospective assessment for DASH, MHQ, and SF-12

**DOI:** 10.1051/sicotj/2016027

**Published:** 2016-10-07

**Authors:** Maurício Vieira de Pádua Maia, Vinícius Ynoe de Moraes, João Baptista Gomes dos Santos, Flávio Faloppa, João Carlos Belloti

**Affiliations:** 1 Disciplina de Cirurgia da mão e membro superior, Universidade Federal de São Paulo, UNIFESP-EPM 04036-001 São Paulo Brazil

**Keywords:** Minimal important difference, DASH, MHQ, SF-12, Hand surgery

## Abstract

*Introduction*: Minimal important difference (MID) score is an important measure for surgical clinical research and impacts on treatment decisions. Our approach considered patient satisfaction as the relevant anchor criteria. The aims of this study were: determine after surgery MID for three relevant questionnaires: Disabilities of the Arm, Shoulder and Hand (DASH), Michigan Hand Questionnaire (MHQ), and Short Form 12 (SF-12); and assess the correlation between these scores and patient reported satisfaction.

*Methods*: Adult patients where surgery was indicated for any hand/wrist conditions. Study was conducted in a teaching hospital, São Paulo, Brazil. Participants responded to DASH, SF-12, MHQ, and a Likert satisfaction scale before and three months after a procedure. Satisfaction was considered as the anchor for determining MID after a procedure. The correlation between satisfaction and the instruments were measured. Two statistical approaches were utilized for determining MIDs and were used for consistency and generalizability purposes. For MID determination, receiver operating curves were utilized and MID cut-offs were followed by sensitivity and specificity measures.

*Results*: Fifty patients were included with no follow-up losses. MID for DASH was 18.8 and 15.4. MID for MHQ was 14.7 for both approaches. Data from SF-12 was not reliable after statistical analyses and demonstrated poor correlation with patient satisfaction. MID for DASH and MHQ were found and demonstrated larger standards than literature-reported patients when surgery was not the main intervention. DASH and MHQ had moderate correlation with patient reported satisfaction. SF-12 MID was not reliable and had poor correlation to patient satisfaction. These data suggests that ambulatory hand surgery patients may have greater expectations regarding improvement than other patients.

## Introduction

Subjective, patient-reported outcomes (PROs) are considered a gold-standard method to assess patient status after an intervention. The development of questionnaires has standardized these measurements and is available for most hand surgery conditions [1].

In upper limb research, Disabilities of the Arm, Shoulder and Hand (DASH) and the Michigan Hand Questionnaire (MHQ) are frequently utilized [2, 3]. For quality-of-life (QoL) assessment, the 12-item Short Form Survey (SF-12) is also ubiquitous [4]. Research indicates that their psychometric properties are adequate for most upper limb conditions [3, 5]. One of the most important psychometric characteristics that change patient care is responsiveness. Responsiveness is defined as the questionnaire ability to detect clinically relevant changes over time [1].

Within this scope, researchers have highlighted the importance of determining what is the minimum difference in questionnaire scores that are relevant to patients, which reflect the concept of minimal important difference (MID). The determination of MID impacts treatment decisions and plays a role in determining sample sizes for prospective research [1, 6].

Hand surgery literature lacks standards for MID, especially when considering heterogeneous samples, such as for patients considered for ambulatory and elective surgery, which is the scope of this study. The data from this study may be adequate as a primer for sample-size calculations, comparative research, and treatment decisions. Some studies have already assessed MID for hand surgery conditions, however included restrict samples and considered MID as secondary objectives. In addition, the considered instruments were not always the most utilized in hand surgery research, such as DASH and MHQ [1, 6].

When evaluating the criteria for determining MID for patient-reported outcomes, most approaches include subjective patient-reported satisfaction as the anchor, as it is considered a simple, reliable, and straightforward measure of perceived improvement after an intervention.

The objectives of this study are twofold: (1) to determine MID for MHQ, DASH, and SF-12 in patients being considered for hand and wrist surgery procedures; and (2) to determine the correlation between these PROs and patient-reported satisfaction.

## Material and methods

The study was approved by the Institutional Ethics Committee (Federal University of São Paulo – São Paulo, Brazil).

### Study design

Prospective comparative, single-center study. Conducted at the hand surgery division of a tertiary university teaching hospital (Hospital São Paulo), São Paulo, Brazil. Patients were enrolled from March to September 2015.

### Patient selection

Adult patients (older than 18 years) regularly attending our offices due to hand/wrist surgery, and traumatic and nontraumatic conditions were potentially assessed for inclusion. The criteria for participant inclusion did not alter the regular office routine, which was a strategy to avoid selection bias. The visit consisted of an assessment by a multidisciplinary hand surgeon in coordinated ambulatory consultation. In this setting, the division’s hand surgery assistants assessed patients independently and they were not influenced by the study purposes and whether or not to indicate surgical procedures.

Patients who had elected to have surgery and fitted the selection criteria were invited to join the study. Informed consent was obtained. Exclusion occurred when patients were not able to follow the study instructions, declared themselves as not being available for prospective follow-up, had complications that resulted in re-operation or any major clinical complication that affected the patient’s health status. Patients with wrist fracture dislocations (such as perilunate dislocations) and nerve injuries were excluded, as this follow-up was not sufficient for MID assessment.

### Outcomes

#### Demographics

The patient’s demographic details such as age, gender, occupation (manual or non-manual), and condition that resulted in surgical intervention were collected.

#### Patient reported questionnaires

For the determination of minimal clinically relevant difference score (MID), three validated questionnaires and a Likert satisfaction scale were applied before (T0) and three months (T1) after a surgical procedure. For all the conditions T1 was considered as sufficient time for ordinary rehabilitation. One of the researchers was responsible for the application of the instruments and did not influence participant responses.

### Disabilities of the Arm, Shoulder and Hand (DASH) questionnaire

Region-specific questionnaire, self-applied. Translated and validated into Brazilian Portuguese [5]. Measures dysfunction of the arm, shoulder, and hand. This evaluation considers activity of both upper limbs globally. In this instrument, lower scores indicate better health [8].

### Michigan Hand Questionnaire (MHQ)

Region-specific questionnaire, self-applied. Indicated for general assessment of all conditions of the hand. Evaluates pain, function, esthetics, and satisfaction. Unlike the DASH questionnaire, it rates separately the left and right hand. Higher scores indicate better hand health [7].

### Short Form 12 (SF-12)

Brief form of the SF-36. Contains mental and physical components. Used as a general QoL measurement tool (health status). Higher scores indicate better health [4].

### Satisfaction

100 mm Likert scale. Patients were instructed to rate their satisfaction by checking on a horizontal line their degree of satisfaction. After evaluation, their measurement was considered as a continuous measure (0–100 mm). Higher scores indicate better satisfaction.

### MID methods

Two methodologies were used to determine MID. Both utilized patient-reported satisfaction changes (from an analog scale) as an anchor. In one approach, based on a measure of dispersion (SD), the external criterion was satisfaction improvement within patients based on 2× baseline standard deviation [9]. The other approach (effect size – ES) was considered by the calculation of satisfaction effect size > 0.8 (change in satisfaction score divided by baseline standard deviation). This criterion is based on Cohen’s effect size theory [10].

From these approaches, data were analyzed with receiver operating characteristic (ROC) curves with the purpose of determining MID for the study instruments. The researcher and a statistical advisor decided which was the best cut-off for MID based on ROC curve data for sensitivity and specificity.

The area under curve (AUC) was also determined for consistency purposes and indicates whether the SD and ES were adequate. We considered inferential analysis (*p*-value) as the criteria for usefulness.

### Statistical methods

All data were analyzed by visual methods in regard to distribution and were considered as parametric. Patient descriptive data were represented as mean and standard deviation (SD) when continuous, and categorical data were exposed with a 95% confidence interval. For all analyses, statistical significance was considered when *p* < 0.05.

Comparisons regarding both methodologies also utilized chi-square (comparison of rates of satisfaction for both criteria). Agreement between MID methodologies was determined by kappa statistics and considered Altman’s criteria (<0.20 = Poor; 0.21–0.40 = Fair; 0.41–0.60 = Moderate; 0.61–0.80 = Good; 0.81–1.00 = Very Good) [11].

Correlation among the instruments was analyzed by Pearson test for correlation. Data was analyzed with SPSS 17 (*SPSS statistics for Windows, Version 17.0. Chicago: SPSS Inc*), except for kappa statistics, which we utilized MiniTab 16 (*Minitab 16 statistical software State College, PA*)*.*

## Results

### Patient demographics

Fifty patients were included, with no follow-up losses for the three month assessment. Patient data are shown in Tables 1 and 2.

**Table 1.** Characteristics of patients admitted for hand/wrist surgery in São Paulo University Hospital, 2015.

Data	Mean (SD)	*N* (%)	Range
Age	42.6 (15.3)		18–73
Male (%)		28 (56)	
Manual workers (%)		20 (41.1)	

**Table 2.** Patient conditions that resulted in surgical treatment.

Condition	*N* (%)
Nerve compression syndromes *	12 (24)
Hand/wrist ganglions	11 (22)
Finger/wrist fractures**	10 (20)
Tendon injuries***	9 (18)
Trigger fingers	5 (10)
Finger ligament injuries****	2 (4)

* Cubital and carpal tunnel syndromes.

** Carpal dislocations were not included.

*** Tendon lacerations and closed injuries.

**** Ulnar collateral thumb ligament lesions.

### MID calculations: methods for determining patient satisfaction

MID satisfaction criteria were considered similar for both methodologies. SD resulted in 86% satisfaction, ES resulted in 72% satisfaction (Chi-square test = 2.95, *p* = 0.08). When comparing both methodologies (ES and SD), it demonstrated fair agreement (Kappa index = 0.59, *p* < 0.001).

### Satisfaction as an anchor: DASH, MHQ, and SF-12 correlations

Baseline data show that DASH and MHQ have moderate correlation with patient-reported satisfaction (Baseline: *r* = 0.58, *p* < 0.001 and *r* = 0.40, *p* = 0.004, respectively; three months: *r* = 0.58, *p* < 0.001 and *r* = 0.64, *p* < 0.001, respectively). DASH was more responsive than MHQ in both periods. SF-12 demonstrated poor agreement with satisfaction in both periods (*r* = 0.21 and 0.002, respectively). Details are shown in Table 3.

**Table 3.** Instrument scores correlation with Satisfaction – baseline and 3-month assessment.

		Dash	MHQ	SF12
Satisfaction (baseline)*	Corr (r)	−0.58	0.40	0.20
	*p*-value	<0.001	0.004	0.161
Satisfaction (3 months)*	Corr (r)	−0.58	0.64	−0.02
	*p*-value	<0.001	<0.001	0.902

* Pearson correlation coefficient.

### MID: cut-offs and accuracy measures

MID sensitivity and specificity calculations (cut-offs derived from ROC curves) are shown in Table 4. SD was superior when compared to ES in the DASH questionnaire analysis (area under curve 0.832 *versus* 0.563). For MHQ analyses, both methods demonstrated adequate statistical significance to detect MID. However, for SF-12, both methods demonstrated poor performance, which indicates that SF-12 MID data may be unreliable. Data supporting MID determinations are additionally provided in supplementary tables.

**Table 4.** MID Calculations – analysis derived from baseline and 3-month assessment.

Instrument	MID (SD)*	MID (ES)*	AUC (SD)**	AUC (ES)**
DASH	18.8 (s: 0.86; e: 0.67)	15.4 (s: 0.43; e: 0.67)	0.832 (*p* = 0.005)	0.563 (*p* = 0.48)
MHQ	14.7 (s: 0.81; e: 1.0)	14.7 (s: 0.81; e: 0.57)	0.900 (*p* = 0.001)	0.748 (*p* = 0.007)
SF-12	2.5 (s: 0.33; e: 0.86)	2.5 (s: 0.36; e: 0.86)	0.543 (*p* = 0.71)	0.593 (*p* = 0.31)

SD = statistical dispersion approach; ES = effect size approach; s = sensitivity; e = specificity; AUC = area under curve.

* Sensitivity and Specificity, derived from Receiver Operating Characteristic curve.

** Area Under the Receiver Operating Characteristic curve.

## Discussion

This study provides important information regarding MID research. To the best of our knowledge this is the first study to consider a heterogeneous sample when determining MID.

Our results are somewhat different from the available research, and we have considered three relevant instruments utilized in routine research and clinical practice [2]. Our results may be utilized for decision making when one is deciding whether a treatment option is adequate, and as a parameter for prospective research, such as when determining a sample-size calculation and/or to check post-hoc statistical power when research is completed [1].

Our data indicates that both MHQ and DASH have negative correlation to patient-reported satisfaction after a surgical procedure. This demonstrates that both instruments have relevant responsiveness and both are appropriate for clinical hand surgery assessment and for research. In our study, however, this was not true for SF-12, since the instrument did not demonstrate similar performance, which is somewhat explained by its broader spectrum and poor correlation with the study’s anchor. From this data, we believe that it may be unnecessary to include QoL instruments as complementary to region- or disease- specific instruments.

Minimal important differences for DASH have been determined for nonoperative conditions [2, 12], and these resulted in a 10-point MID. Our results may have been inflated due to the fact that surgery was the only treatment, which possibly creates a more demanding environment and higher patient expectations.

For MHQ, the data are similar to a previous study [13], however, two important aspects should be noted: the possibility of an important ceiling effect [14] and the absence of a MHQ domain-by-domain assessment of MID. We also came to the same opinion as a previous study that MHQ calculations are more arduous and demonstrated no better performance when compared to DASH [15].

Our study has some limitations, including a small sample size and the absence of longer periods of follow-up. Advantages are the inclusion of different conditions and assessment of three instruments, which broadens the external validity of our data.

Methodologies utilized to determine MID have not reached common standards and our methodology is similar to findings in several other research studies [1–6]. An anchor-based approach has the disadvantage of considering only arbitrary (researcher-based) criteria, however, it is more adequate than an isolated distribution-based approach, especially when considering different conditions and small sample sizes. In an effort to increase the study’s internal validity we considered two approaches, one of which was derived from a distribution-based methodology (SD), which has been recommended in the literature [14]. However, our analysis could not avoid the inherent subjective decision for cut-off determinations.

In conclusion, minimal important differences for DASH were found to be 18.8 (SD) and 15.4 (ES). MHQ MID was found to be 14.7 for both approaches. SF-12 MID was not reliable and had poor correlation to patient satisfaction. MID for DASH and MHQ demonstrated larger standards and as such for literature-reported patients where surgery was not the main intervention. This data suggests that ambulatory hand surgery patients may have greater expectations regarding improvement than other patients. Researchers should consider the possibility of considering larger effect sizes when delineating surgery-based trials.

## Conflict of interest

The author(s) declare no conflict of interest in relation with this paper.
